# Selective Serotonin Reuptake Inhibitors and Symptoms of Depression in Patients on Chronic Hemodialysis: A Systematic Review

**DOI:** 10.3390/jcm13113334

**Published:** 2024-06-05

**Authors:** Maurizio Bossola, Ilaria Mariani, Manuela Antocicco, Gilda Pepe, Anna Petrosino, Enrico Di Stasio

**Affiliations:** 1 Servizio Emodialisi, Dipartimento di Scienze Mediche e Chirurgiche, Università Cattolica del Sacro Cuore, 00168 Rome, Italy; 2 Policlinico Universitario Fondazione Agostino Gemelli IRCCS, 00168 Rome, Italy; 3 Dipartimento Scienze dell’Invecchiamento, Neurologiche, Ortopediche e Della Testa-Collo, 00168 Rome, Italy; 4 Divisione Chirurgia d’Urgenza, Dipartimento di Scienze Mediche e Chirurgiche, Università Cattolica del Sacro Cuore, 00168 Rome, Italy; 5 Dipartimento di Scienze Biotecnologiche di Base, Cliniche Intensivologiche e Perioperatorie, Università Cattolica del Sacro Cuore, 00168 Rome, Italy

**Keywords:** hemodialysis, depression, selective serotonin reuptake inhibitors, SSRI

## Abstract

**Objective:** The use of selective serotonin reuptake inhibitors (SSRIs) is common among hemodialysis patients who receive treatment for depression. However, studies on the efficacy of SSRIs in patients on chronic hemodialysis are few and have led to conflicting results. The present systematic review aims to evaluate, in randomized, controlled studies (RCSs), the efficacy of SSRI administration in reducing symptoms of depression in patients on chronic hemodialysis when compared with placebo or psychological interventions. **Method**: Research was run on December 2023 in the following databases: Ovid MEDLINE (1985 to present); Ovid EMBASE (1985 to present); Cochrane Library (Wiley); and PubMed (1985 to present). The primary outcome was the frequency and severity of the symptoms of depression assessed through the Beck Depression Inventory (BDI) or the Hamilton Depression Rating Scale (HAMD). The secondary outcome was the prevalence of adverse events. **Results**: Seven studies totaling 433 patients were included. The number of patients in each individual study ranged from 13 to 120. The length of studies ranged from 8 weeks to 6 months. Heterogeneous data precluded informative meta-analysis. Three studies compared sertraline with a placebo. Of these, two demonstrated that sertraline was better than the placebo in reducing the symptoms of depression while one showed no statistically significant differences between sertraline and the placebo. One study, comparing fluoxetine with a placebo showed that the symptoms of depression did not differ significantly at 8 weeks. In another study, escitalopram administration led to a significantly greater reduction in the Hamilton Depression Rating Scale score compared to a placebo, as well as in the Hamilton Anxiety Rating Scale score. In one study, citalopram and psychological interventions were both effective in reducing the symptoms of depression and anxiety and, in another study, sertraline was modestly more effective than CBT at 12 weeks in reducing the symptoms of depression. **Conclusions**: SSRIs may be effective in reducing the symptoms of depression in patients on chronic hemodialysis. SSRI administration, at the dosage used in the studies included in the present systematic review, seems safe in most hemodialysis patients. However, the paucity of studies and the limited number of patients included in the trials may suggest that further randomized, controlled studies are needed to determine if SSRIs may be used routinely in daily clinical practice in such a population.

## 1. Introduction

Depression is common in end-stage renal disease (ESRD) patients on chronic hemodialysis, potentially affecting up to 60–80% of patients and being significantly more frequent than in the age-matched general population [1,2,3,4,5]. 

Depression is associated with impaired quality of life, higher frequency of hospitalization, and lower adherence to treatments [1,2,3,4,5]. In addition, an increased risk of all-cause mortality in adults with depression and ESRD has been found consistently; a systematic review of 22 studies involving 83,381 participants demonstrates a hazard ratio of 1.59 (95% CI: 1.35–1.87) [3]. 

There are no established guidelines for treating depression in adults with ESRD on chronic hemodialysis. Less than 25% of those on hemodialysis who are diagnosed with moderate or severe depression undergo treatment [6,7,8,9,10,11,12]. 

The use of selective serotonin reuptake inhibitors (SSRIs) is common among hemodialysis patients who receive a treatment for depression. In the Dutch study of van Osten et al., antidepressant prescription was higher in chronic kidney disease (CKD) patients (5.6% versus 2.8%; *p* < 0.001) and in hemodialysis patients (5.3% versus 3.0%; *p* < 0.001) than in controls [11]. In addition, SSRIs were among the most frequent medications among patients who were prescribed antidepressant medications (55% in CKD patients and 61% in dialysis patients) [11]. The 2020 USRDS report shows that the percentage of patients undergoing hemodialysis (HD) using SSRIs in the USA is 21.5% [12]. In a small group of UK patients, 11% of them used antidepressants, and SSRIs in particular [7]. Finally, in a cross-sectional analysis of the Dialysis Outcomes and Practice Patterns Study II cohort, including hemodialysis patients from 12 different countries, Lopes et al. report antidepressant use of ~13% [8]. 

Nevertheless, studies on the efficacy of SSRIs in patients on chronic hemodialysis are few and have led to conflicting results [13,14,15,16,17,18,19,20].

Thus, the question is if this high use of SSRIs in patients on chronic hemodialysis in routine clinical practice is justified by the evidence of the data of adequate clinical trials.

The present systematic review aims to evaluate the efficacy of SSRI administration in reducing symptoms of depression in patients on chronic hemodialysis.

According to the PICOS criteria, we analyzed: population: end-stage renal disease patients on chronic hemodialysis; intervention: SSRI administration orally; comparison: no intervention or psychological intervention; outcome: symptoms of depression assessed through the Beck Depression Inventory or other scale; study: systematic review of randomized controlled studies. The primary outcome of the review is to determine the difference between oral SSRI administration and no intervention or other interventions in reducing symptoms of depression.

## 2. Methods

This analysis was prospectively registered on the International Prospective Register of Systematic Reviews in Health and Social Care (PROSPERO, ID number CRD42023414581). 

### 2.1. Eligibility Criteria

Studies were eligible for inclusion if they were published in a peer-reviewed journal and met the following inclusion criteria: (1) primary research studies in adult patients (over 18 years of age); (2) patients with end-stage renal disease on chronic hemodialysis for at least 6 months; (3) compared oral SSRI administration with no intervention or with other interventions in terms of reduction in symptoms of depression assessed through the Beck Depression Inventory (BDI), the Hamilton Depression Rating Scale (HAMD), the Quick Inventory of Depressive Symptomatology (QIDS-C), the Hospital Anxiety and Depression Scale (HADS), the Montgomery Asberg Depression Rating Scale (MADRS), or the Brief Symptom Inventory (BSI); and (4) randomized, controlled studies. We excluded studies on pediatric patients, pre-dialysis CKD patients, acute kidney injury patients, ESRD patients with other renal replacement therapy modalities such as peritoneal dialysis and transplant. 

### 2.2. Search Strategy

A medical librarian performed comprehensive research to identify studies that compared the effect of SSRIs with a placebo or with psychological interventions on symptoms of depression in patients on chronic hemodialysis. Research was run on December 2023 on the following databases: Ovid MEDLINE (1985 to present); Ovid EMBASE (1985 to present); Cochrane Library (Wiley); PubMed (1985 to present). Search terms and mesh headings included “hemodialysis/haemodialysis” AND (“major depression” OR “depression” OR “symptoms of depression” OR “depressive symptoms”) AND “Beck Depression Inventory” AND “BDI” AND “Hamilton depression scale” AND “Hamilton Depression Rating Scale” AND “HAMD” AND “Quick Inventory of Depressive Symptomatology-Clinician rated” AND “QIDS-C” AND “Hospital Anxiety and Depression Scale” AND “HADS” AND “Montgomery Asberg Depression Rating Scale” AND “MADRS” AND “Brief Symptom Inventory” AND “BSI” AND “depression scale” AND “sertraline” AND “selective serotonin reuptake inhibitors” AND “SSRI” AND “citalopram” AND “escitalopram” AND “fluoxetine” AND “fluvoxamine” AND “paroxetine” AND “dapoxetine” AND “vortioxetine” AND “ antidepressant” AND “psychological intervention” AND “ cognitive behavioral therapy” AND “psychological training”. This review followed the Preferred Reporting Items for Systematic Reviews and Meta-Analyses (PRISMA) reporting guideline.

### 2.3. Data Extraction

Database research screening and exclusion of duplicated results was performed by a qualified medical librarian. Two investigators screened the initial search results for inclusion and performed data extraction independently. Disagreements were resolved by a third author, who also checked the extracted data for accuracy. Full text for the selected studies was pulled for a second round of eligibility screening. Reference lists of articles were also searched to identify other relevant studies

### 2.4. Quality Assessment and Risk of Bias

The quality of reporting for each study was performed by two researchers using the Quality Assessment Tool of Controlled Intervention Studies of the National Institutes of Health [21]. 

### 2.5. Outcomes

The primary outcomes were the frequency and severity of the symptoms of depression assessed through any validated depression assessment tool. The secondary outcome was the frequency of adverse events. 

## 3. Results

### 3.1. Literature Search 

**Figure 1:**
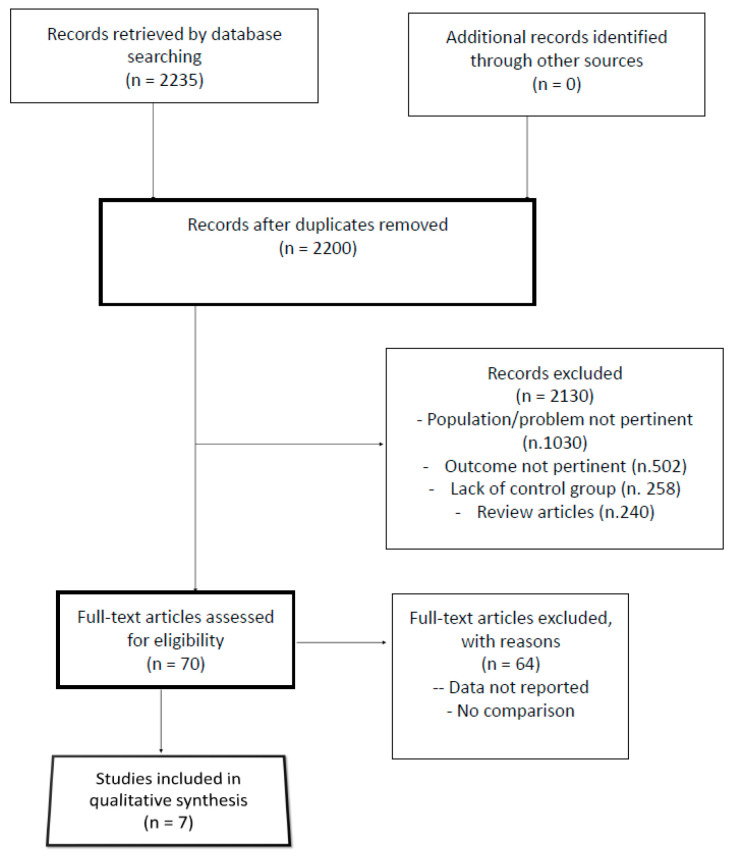
Preferred Reporting Items for Systematic Reviews and Meta-Analyses (PRISMA) flowchart of our analysis.

Two thousand, two hundred and thirty-five records were identified through database and hand searches. Of these, 35 were excluded based on duplicated data, and 2200 titles and abstracts were evaluated. Seven articles were fully assessed for eligibility and included in the investigation results [13,14,15,16,17,18,19,20]. The PRISMA flow diagram outlining the study selection process is available in Figure 1.

### 3.2. Characteristics of the Studies Included

Overall, 433 patients were included. The number of patients in each individual study ranged from 13 to 125. The length of studies ranged from 8 weeks to 6 months and their descriptions are presented in Table 1. All studies were prospective, randomized, and controlled. Three studies compared the use of sertraline vs. placebo [14,18,20], one study sertraline vs. cognitive behavioral therapy (CBT) [17], one study compared fluoxetine vs. placebo [13], one study escitalopram vs. placebo [19], and one study compared citalopram vs. psychological training [15]. The depression assessment tools were the Beck Depression Inventory (BDI) in three studies [13,14,18], the Hospital Anxiety and Depression Scale (HADS) in one study [15], the Hamilton Depression Rating Scale (HAMD) in two studies [19,20], and the Quick Inventory of Depressive Symptomatology-Clinician Rated (QIDS-C) in one study [17] (Table 1).

### 3.3. Efficacy of Interventions on Symptoms of Depression

The efficacy of SSRIs on symptoms of depression is shown in Table 2. Three studies compared sertraline with a placebo [14,18,20]. Of these, two demonstrated that sertraline was better than the placebo in reducing the symptoms of depression [18,20], while one showed no statistically significant differences between sertraline and the placebo [14]. In particular, in the study of Taraz et al., in patients receiving sertraline (50 mg/daily for 2 weeks and 100 mg/daily for the following 10 weeks) the Beck Depression Inventory (BDI-II) score decreased from 29 ± 13 at baseline to 15 ± 5. 5 at 12 weeks (*p* < 0.001), while remaining essentially unchanged in the placebo group (23 ± 11 to 22.5 ± 9), the changes from baseline to week 12 of the study being −11.3 ± 5.8 and −0.5 ± 5, respectively (*p* < 0.001) [18]. The recent study of Zhang et al. demonstrated that after 12 weeks of treatment the Hamilton Depression Rating Scale (HAMD) score of patients receiving sertraline (initial dose 25–50 mg/daily, then the dose was adjusted based on patient’s response) significantly decreased compared to the placebo group, and that of the 62 patients included in the sertraline group, 10 and 50 achieved complete and partial remission of the symptoms, respectively [20]. In the study of Friedli et al., of the 30 patients originally randomized to sertraline (50 mg/daily with eventual increase to 100 mg/daily at month 2 and/or 4) or to a placebo, only 21 completed the study, 8 in the sertraline group and 13 in the placebo group, due to a large number of dropouts for adverse events (n = 7 in the sertraline group and none in the placebo group; *p* = 0.04). With regard to the outcomes, the mean change in the Montgomery Asberg Depression Rating Scale (MADRS) score over the 6 months of the study was −14.5 (95% CI: −20.2 to −8.8) in the sertraline group and −14.9 (95% CI: −18.4 to −11.5) in the placebo group (*p* > 0.05) and changes in BDI-II scores also were similar at −15.7 (95% CI: −24.3 to −7.1) in the sertraline group and −13.0 (95% CI: 19.6 to −6.4) in those on the placebo (*p* > 0.05) [14].

The small study of Blumenfield et al., comparing fluoxetine (n = 6 patients) with a placebo (n = 7 patients) at a dosage of 20 mg/daily, showed that the scores of the BDI, Brief Symptom Inventory, and MADRS did not differ significantly at 8 weeks [13]. In the study of Yazici et al., escitalopram administration led to a significantly greater reduction in the Hamilton Depression Rating Scale (HAMD) score (from 27 [7–43] to 10.5 [4–35]; *p* = 0.001) than administration of a placebo (from 31 [14–39] to 28 [7–35]; *p* > 0.05), as was also seen for the Hamilton Anxiety Rating Scale (HAMA) score (from 22.8 [8–46] to 13.5 [3–43]; *p* < 0.0001; and from 27 [16–40] to 29.5 [13.41]; *p* > 0.05, respectively) [19]. 

Two studies compared SSRIs with psychological interventions. The study of Hosseini et al. evaluated the effect of citalopram administration and psychological training on the scores of depression and anxiety. Both led to a significant decrease in the patients’ mean depression score (from 9.42 ± 3.11 to 6.2 ± 4.1; *p* = 0.001; and from 9.5 ± 3.4 to 7.3 ± 4.8; *p* = 0.04, respectively), anxiety score (from 10 ± 3.1 to 8.1 ± 5.6; *p* = 0.048; and from 9.1 ± 2 to 7.1 ± 4.1; *p* = 0.03, respectively), and total HADS score (from 19.4 ± 4.7 to 14.4 ± 8.8; *p* = 0.002; and from 18.6 ± 5 to 15.1 ± 6.1; *p* = 0.045), without significant difference between the two groups (Hosseini et al., 2021). In the study of Mehrotra et al., compared with cognitive behavioral therapy (CBT), sertraline treatment resulted in a moderately higher reduction in the QIDS-C (Quick Inventory of depressive symptomatology-clinical related) scores at 12 weeks (from 10.9 ± 4.9 to 5.9 ± 4.5) than CBT (from 12.2 ± 5.1 to 8.1 ± 5.1), with an effect estimate vs. CBT of −1.84 [CI, −3.54 to −0.13]; *p* = 0.035) [17]. 

### 3.4. Adverse Events

Adverse events of each study are reported in Table 3. In two studies, the frequency of nausea was significantly higher in patients receiving sertraline than in those receiving the placebo [15,21], while in two other studies it was similar [13,19]. The frequency of the other adverse events did not differ significantly between the groups in all studies [13,14,15,16,17,18,19,20]. Dropout rates for adverse events ranged from 6.6% to 30%, but they did not differ significantly between treatment and control groups [13,14,18,19,20]. 

### 3.5. Quality Assessment

Table 4 shows that the overall score of randomized, controlled studies comparing SSRI vs. placebo or SSRI vs. other treatments ranged from 9 to 13, being 9 in one study [15], 10 in three studies [13,14,19], 11 in one study [20], and 13 in two studies [17,18]. 

## 4. Discussion

The present systematic review shows that in three studies, including 216 patients, SSRIs significantly improved the symptoms of depression when compared with a placebo [18,19,20], while in two studies, including 34 patients, they did not [13,14]. 

The present systematic review also shows that in one study citalopram and psychological interventions were both effective in reducing the symptoms of depression and anxiety [17], and in another study that sertraline was modestly more effective than CBT at 12 weeks in reducing the symptoms of depression [15]. 

The recent meta-analysis by Nadort et al., comparing two studies on sertraline versus placebos in patients on chronic HD, showed an effect size of −0.57, with a large confidence interval (−6.17; 5.02) and a high heterogeneity (I^2^ = 71%, X^2^ = 0.2474, *p* = 0.06) [9]. The systematic review of Chopra et al., that included three studies comparing SSRI vs. placebo in patients on chronic hemodialysis, concluded that there was not sufficient evidence to draw definitive conclusions about the effectiveness of SSRIs in treating depression in such patients [6]. 

Interestingly, a recent randomized clinical trial that included 201 patients with stage 3, 4, or 5 non-dialysis-dependent chronic kidney disease and at least moderate depressive symptoms, demonstrated that the use of sertraline vs. placebo did not result in a statistically significant difference in symptom improvement over 12 weeks [22]. 

Notably, although some studies have shown a statistically significant reduction in the symptoms of depression with the use of SSRIs in patients on chronic hemodialysis, it remains to be defined what constitutes a clinically significant finding in routine clinical practice in such patients. 

Patients on chronic hemodialysis are at higher risk for SSRI adverse events due to the potential accumulation of toxic metabolite effects in the setting of a reduced glomerular filtration rate [22]. Indeed, the present systematic review shows that, in most studies, the frequency of adverse events was not significantly higher in patients receiving SSRIs than in those receiving a placebo or a psychological intervention, with the exception of nausea that in two studies out of four was significantly higher with sertraline than with the placebo. However, the paucity of patients studied and the relatively short length of the follow-up of the trials may have masked the occurrence of some of these adverse events. In fact, a large-population study showed strong evidence that the excess risk of gastrointestinal bleeding increased substantially as renal function declined, ranging from 2.0/1000 person-years among patients with no CKD diseases to 7.9/1000 person-years among patients with CKD stage 4/5 [23]. In addition, by the analysis of the USRDS, Vangala et al. found that SSRI use among hemodialysis patients was associated with increased hip fracture risk [adjusted OR (odds ratio), 1.25 [95% CI: 1.17–1.35]] and that the association between hip fracture events and SSRI use was also seen in the examination of new short-term use (adjusted OR: 1.43; 95% CI: 1.23–1.67) [24]. Finally, all available SSRIs can prolong the QT interval [25] and there is evidence among patients on chronic hemodialysis that citalopram or escitalopram administration is associated with an increased risk of sudden cardiac death with respect to other SSRIs [26]. 

The present review has some limitations. First, the sample size of many of the included trials was small and, overall, only 433 patients were studied. Second, the length of the studies was extremely varied and short, ranging from two months to six months. Third, dropout rates were high in most studies. Fourth, only two studies appropriately diagnosed depression by using a psychiatric interview. Fifth, all studies evaluated the outcome depression using depression scales that are known to overestimate the prevalence of depression in patients on chronic hemodialysis for the concomitant high frequency of other symptoms such as anorexia and fatigue [10]. Sixth, pharmacokinetic data of antidepressants were unavailable in the original studies. Seventh, a meta-analysis was not feasible due to the heterogeneity of the included studies. Finally, only a tiny number and type of antidepressants has been tested thus far in this population;

In conclusion, the evidence in the current literature suggests that SSRIs may be effective in reducing the symptoms of depression in patients on chronic hemodialysis. SSRI administration, at the dosage used in the studies included in the present systematic review, seems safe in most hemodialysis patients. However, the limitations of the studies detailed above suggest that further randomized, controlled studies are needed to determine if SSRIs may be used routinely in daily clinical practice in such a population.

## Figures and Tables

**Table 1:** Characteristics of studies on the effect of SSRIs on depression in patients on chronic hemodialysis. Data are expressed as mean ± SD. CBT, cognitive behavioral therapy; BDI, Beck Depression Inventory; BSI, Brief Symptom Inventory; MADRS, Montgomery Asberg Depression Rating Scale; HADS, Hospital Anxiety and Depression Scale (HADS); HAMD, Hamilton Depression Rating Scale; QIDS-C, Quick Inventory of Depressive Symptomatology—Clinician Rated; RCT, randomized controlled trial; NR, not reported.

	Country	Type of Study	Number of Patients (Intervention: Placebo)	Age (yrs)	Sex	Race	Intervention	Duration	SSRI Dosage
Blumenfield et al., 1997 [13]	USA	RCT	13 Fluoxetine: n = 6 Placebo: n = 7	NR	NR	NR	Fluoxetine vs. placebo	8 weeks	20 mg/daily
Yazici et al., 2012 [19]	Turkey	RCT	58 Escitalopram: n = 30 Placebo: n = 28	51	Males 52%	NR	Escitalopram vs. placebo	8 weeks	20 mg/daily
Taraz et al., 2013 [18]	Iran	RCT	43 Sertraline: n = 21 Placebo: n = 22	62	Males 58%	NR	Sertraline vs. placebo	12 weeks	50 mg/daily for 2 weeks and 100 mg/daily for the remaining 10 weeks
Friedli et al., 2017 [14]	UK	RCT	21 Sertraline: n = 8 Placebo: n = 13	59	Males 76.6%	NR	Sertraline vs. placebo	6 months	50 mg/daily with eventual increase
Zhang et al., 2024 [20]	China	RCT	125 Sertraline: n = 62 Placebo: n = 63	59	Males 52.8%	NR	Sertraline vs. placebo	12 weeks	25–50 mg/daily, then dose adjusted based on patient’s score
Hosseini et al., 2012 [15]	Iran	RCT	44 Citalopram: n = 22 Psychological intervention: n = 22		Males 43%	NR	Citalopram vs. psychological training	12 weeks	20 mg/daily
Mehrotra et al., 2019 [17]	USA	RCT	120 Sertraline: n = 60 CBT: n = 60		Males 57%	NR	Sertraline vs. CBT	12 weeks	25 mg/daily in the first week and 50 mg/daily in the second week. Then, dosage was titrated every 2 weeks for the following 4 weeks until 200 mg/daily, and then, maintained for the following 6 weeks

**Table 2:** Studies on the effect of SSRIs on depression in patients on chronic hemodialysis: Outcomes. Data are expressed as mean ± SD. CBT, cognitive behavioral therapy; BDI, Beck Depression Inventory; BSI, Brief Symptom Inventory; MADRS, Montgomery Asberg Depression Rating Scale; HADS, Hospital Anxiety and Depression Scale (HADS); HAMD, Hamilton Depression Rating Scale; QIDS-C, Quick Inventory of Depressive Symptomatology—Clinician Rated.

	Intervention	Measurement	Outcome
Blumenfield et al., 1997 [13]	Fluoxetine vs. placebo	BDI; BSI; MADRS	BDI, BSI, and MADRS similar in the 2 groups.
Yazici et al., 2012 [13]	Escitalopram vs. placebo	HAMD	Escitalopram led to a significantly greater reduction in HAMD score (from 27 [7–43] to 10.5 [4–35]) compared to placebo (from 31 [14–39] to 28 [7–35]) (*p* = 0.001).
Taraz et al., 2013 [18]	Sertraline vs. placebo	BDI-II	BDI-II score decreased from 29 ± 13 at baseline to 15 ± 5. 5 at 12 weeks (*p* < 0.001) in the sertraline group while remaining essentially unchanged in the placebo group (23 ± 11 to 22.5 ± 9).
Friedli et al., 2017 [14]	Sertraline vs. placebo	BDI-II; MADRS	Mean change in MADRS score over the 6 months of the study was −14.5 [95% CI]−20.2 to −8.8] in the sertraline group and −14.9 [95% CI: −18.4 to −11.5] in the placebo group. Changes in BDI-II scores were similar at −15.7 [95% CI: −24.3 to −7.1] in the sertraline group and −13.0 [95% CI: 19.6 to −6.4] in those on the placebo. No statistically reliable differences between the groups.
Zhang et al., 2024 [20]	Sertraline vs. placebo	HAMD	At 12 weeks, HAMD scores of patients in the treatment group significantly decreased compared to before treatment, whereas there was no significant change in the placebo group. At week 12, HAMD score was 10 in the sertraline group and 18 in the placebo group (*p* < 0.001).
Hosseini et al., 2012 [15]	Citalopram vs. psychological training	HADS	Both led to a significant decrease in the patients’ depression score (from 9.42 ± 3.11 to 6.2 ± 4.1; *p* = 0.001; and from 9.5 ± 3.4 to 7.3 ± 4.8; *p* = 0.04, respectively), anxiety score (from 10 ± 3.1 to 8.1 ± 5.6; *p* = 0.04; and from 9.1 ± 2 to 7.1 ± 4.1; *p* = 0.03, respectively), and total HADS score (from 19.4 ± 4.7 to 14.4 ± 8.8; *p* = 0.00; and from 18.6 ± 5 to 15.1 ± 6.1; *p* = 0.045, respectively), without significant difference between the two groups.
Mehrotra et al., 2019 [17]	Sertraline vs. CBT	QIDS-C	Sertraline treatment resulted in greater reduction in the QIDS-C score at 12 weeks (from 10.9 ± 4.9 to 5.9 ± 4.5) than CBT (from 12.2 ± 5.1 to 8.1 ± 5.1), with an effect estimate vs. CBT of −1.84 [CI, −3.54 to −0.13]; *p* = 0.035.

**Table 3:** Studies on the effect of SSRIs on depression in patients on chronic hemodialysis: adverse events. NR, not reported.

		Hypotension	Anorexia	Nausea	Vomiting	Headache	Insomnia	Somnolence	Dizziness	Diarrhea	Xerostomia	Sexual Disturbances	Major Bleeding
Blumenfield et al., 1997 [13]	Fluoxetine (n = 6) vs. placebo (n = 7)	4/6 1/7 *p* = 0.102		5/6 2/7 *p* = 0.102	3/6 3/7 *p* = 1.000	3/6 0/7 *p* = 0.069	2/6 1/7 *p* = 0.559	NR	NR	NR	0/6 1/7 *p* = 1.000	NR	NR
Yazici et al., 2012 [19]	Escitalopram (n = 28) Vs. placebo (n = 30)			5/28 2/30 *p* = 0.479		5/28 4/30 *p*= 0.912	4728 2/30 *p* = 0.731	2/28 2/30 *p* = 0.656	4/28 4/30 *p* = 0.778	2/28 1/30 *p* = 0.951	NR	4/28 0/30 *p* = 0.139	NR
Hosseini et al., 2012 [15]	Citalopram vs. psychological training	Adverse events did not present with Citalopram											
Taraz et al., 2013 [18]	Sertraline (n = 21) vs. placebo (n = 22)	NR	2/21 4/22 *p* = 0.413	7/21 3/22 *p* = 0.033	6/21 4/22 *p* = 0.255	4721 2/22 *p* = 0.412	NR	NR	5/21 3/22 *p* = 0.126	NR	NR	2/21 1/22 *p* = 0.607	NR
Friedli et al., 2017 [14]	Sertraline (n = 15) vs. placebo (n = 15)	9 adverse events in each group but 7 dropouts for severe adverse events in the sertraline group											
Mehrotra et al., 2019 [19]	Sertraline (n = 69) vs. CBT (n = 60)	NR	NR	15/60 7/60 *p* = 0.09		NR	NR	NR	NR	NR	4/28 4/30 *p* = 0.778	NR	1/60 1/60 *p* = 1.000
Zhang et al., 2024 [20]	Sertraline (n = 62) vs. placebo (n = 63)	NR	NR	12/62 4/63 *p* = 0.030	NR	5/62 4/63 *p* = 0.980	NR	NR	6/62 6/63 *p* = 0.977	4/62 5/62 *p* = 1.000	NR	4/62 3/63 *p* = 0.983	NR

**Table 4:** Quality analysis of prospective, randomized, controlled studies on the effect of SSRIs on symptoms of depression. Y = yes; N = no; NR = not reported. Legend: 1. Was the study described as randomized, a randomized trial, a randomized clinical trial, or an RCT? 2. Was the method of randomization adequate (i.e., use of randomly generated assignment)? 3. Was the treatment allocation concealed (so that assignments could not be predicted)? 4. Were study participants and providers blinded to treatment group assignment? 5. Were the people assessing the outcomes blinded to the participants’ group assignments? 6. Were the groups similar at baseline on important characteristics that could affect outcomes (e.g., demographics, risk factors, co-morbid conditions)? 7. Was the overall drop-out rate from the study at endpoint 20% or lower of the number allocated to treatment? 8. Was the differential drop-out rate (between treatment groups) at endpoint 15 percentage points or lower? 9. Was there high adherence to the intervention protocols for each treatment group? 10. Were other interventions avoided or similar in the groups (e.g., similar background treatments)? 11. Were outcomes assessed using valid and reliable measures, implemented consistently across all study participants? 12. Did the authors report that the sample size was sufficiently large to be able to detect a difference in the main outcome between groups with at least 80% power? 13. Were outcomes reported or subgroups analyzed prespecified (i.e., identified before analyses were conducted)? 14. Were all randomized participants analyzed in the group to which they were originally assigned, i.e., did they use an intention-to-treat analysis?

Authors	1	2	3	4	5	6	7	8	9	10	11	12	13	14	Overall Score
Blumenfield et al., 1997 [13]	Y	Y	Y	Y	Y	Y	Y	N	NR	NR	Y	N	Y	Y	10
Yazici et al., 2012 [19]	Y	Y	Y	NR	NR	Y	Y	Y	NR	Y	Y	NR	Y	Y	10
Hosseini et al., 2012 [15]	Y	NR	NR	N	NR	Y	Y	Y	Y	NR	Y	Y	Y	Y	9
Taraz et al., 2013 [18]	Y	Y	Y	Y	NR	Y	Y	Y	Y	Y	Y	Y	Y	Y	13
Friedli et al., 2017 [14]	Y	Y	Y	Y	Y	Y	N	N	Y	Y	Y	N	Y	Y	10
Mehrotra et al., 2019 [17]	Y	Y	Y	Y	Y	Y	Y	Y	Y	NR	Y	Y	Y	Y	13
Zhang et al., 2024 [20]	Y	Y	Y	NR	NR	Y	Y	Y	Y	Y	Y	NR	Y	Y	11
